# A step forward in botanical exploration with three new Polytrichaceae records from Tibet, China

**DOI:** 10.3897/BDJ.12.e133971

**Published:** 2024-10-03

**Authors:** Wei Zhao, Zhaoming Wang, Lina Zheng, Gaowa Naren, Qiang He, Wei Han

**Affiliations:** 1 M-Grass Ecological Environment (Group) Co., Ltd, Hohhot, China M-Grass Ecological Environment (Group) Co., Ltd Hohhot China; 2 State Key Laboratory of Plant Diversity and Specialty Crops/State Key Laboratory of Systematic and Evolutionary Botany, Institute of Botany, Chinese Academy of Sciences, Beijing, China State Key Laboratory of Plant Diversity and Specialty Crops/State Key Laboratory of Systematic and Evolutionary Botany, Institute of Botany, Chinese Academy of Sciences Beijing China; 3 School of Resources & Chemical Engineering, Sanming University, Sanming, China School of Resources & Chemical Engineering, Sanming University Sanming China

**Keywords:** bryophytes, Tibet, China, new record, checklist

## Abstract

**Background:**

According to the Species Catalogue of China, Tibet, China, has more than 1,000 species of bryophytes, showing its remarkably high biodiversity. Amongst them, the Polytrichaceae is one of the largest family, with six genera and 25 species, one subspecies and two varieties reported previously.

**New information:**

Based on a field survey and specimen identification, the following species have been newly recorded in the bryoflora of Tibet, namely *Oligotrichumobtusatum* Broth., *Pogonatumcontortum* (Menzies ex Brid.) Lesq. and *Polytrichumsphaerothecium* (Besch.) Müll. Hal.

## Introduction

The Bryophyte Flora of Tibet has been documented to include 62 families, 254 genera and 754 species, including five subspecies, 53 varieties and three forms (Li 1985). Despite recent additions to the regional bryophyte records, there still remains a significant gap in the comprehensive exploration of the bryophyte flora in Tibet (Ma et al. 2018a, Ma et al. 2018b, Ma et al. 2018c). According to the Species Catalogue of China, the number of moss species in Tibet alone is estimated to exceed 1,000 (Wang et al. 2018).

The family Polytrichaceae Schwägr. represents a significant group of mosses with a global distribution that includes 18 genera and approximately 220 species (Frey et al. 2009). In China, this family is represented by seven genera and 51 species, while Tibet specifically hosts six genera and 25 species, one subspecies and two varieties (Wu and Jia 2004, Isuru et al. 2018, Wang et al. 2018). Members of the family are characterised by relatively large and robust plants that are typically erect and rigid, forming dense tufts or growing scattered or gregariously, resembling conifer seedlings, with well-developed central strand in the stem cross-section. Besides, leaves usually have broad sheathing at the base, with single and rather broad costa and numerous lamellae, usually longitudinally arranged on the ventral surface of the costae, sometimes lamellae or spines present on the dorsal surface. Species in this group are generally dioecious, though some are monoecious and typically feature relatively large sporophytes with rigid setae, hairy calyptrae and nematodontous peristomes with 32–64 teeth. Moreover, the plants have terminal discoid perigonia and capsules that are mostly oblong-ovoid to cylindrical or 4-6-angular, with a single lingulate peristome (Wu et al. 2005, Bell et al. 2021, Merrill 2024).

This study conducted an investigation of wild plant resources in Tibet, China, revealing the presence of five genera and 12 species of Polytrichaceae. Amongst them, three were identified as new records. Detailed descriptions and illustrations of the species are provided and a checklist is also given.

## Materials and methods

All the specimens, examined in this study, were collected from Tibet in China at 2018 and deposited in the Herbarium (PE), Institute of Botany, the Chinese Academy of Sciences, Beijing. The newly-collected specimens of Polytrichaceae were identified by Y. Jia and Q. He from the Institute of Botany, Chinese Academy of Sciences. Photographs were taken using a Leica DM 4000 B LED microscope (Leica Microsystems, Wetzlar, Germany). Illustrations of *O.obtusatum*, *P.contortum* and *P.sphaerothecium* were respectively drawn by Z.D. Yang from Kunming Institute of Botany, Chinese Academy of Sciences, P.C. Wu and M.S. Guo from Institute of Botany, the Chinese Academy of Sciences.

## Taxon treatments

### Oligotrichum obtusatum

Broth.

6D32B4C1-F51C-5DA4-BCB3-02E853255388

#### Materials

- **Type status:** Other material. **Occurrence:** catalogNumber: PE2155874; recordNumber: 12176; recordedBy: Q. He; occurrenceID: D9CB2D58-4FEC-5582-940F-61363733E69C; **Taxon:** order: Polytrichales; family: Polytrichaceae; genus: Oligotrichum; **Location:** continent: Asia; country: China; countryCode: China/CN; stateProvince: Tibet; county: Jilong; municipality: Jilong; locality: Latola; verbatimLocality: Shigatse; verbatimElevation: 3954 m; verbatimCoordinates: 28º23′26"N, 85º24′25"E; **Identification:** identifiedBy: Y. Jia and Q. He; **Event:** year: 2018; month: 6; day: 17; habitat: On the roadside soil surface; **Record Level:** language: cn

#### Description

**Figure 1.**
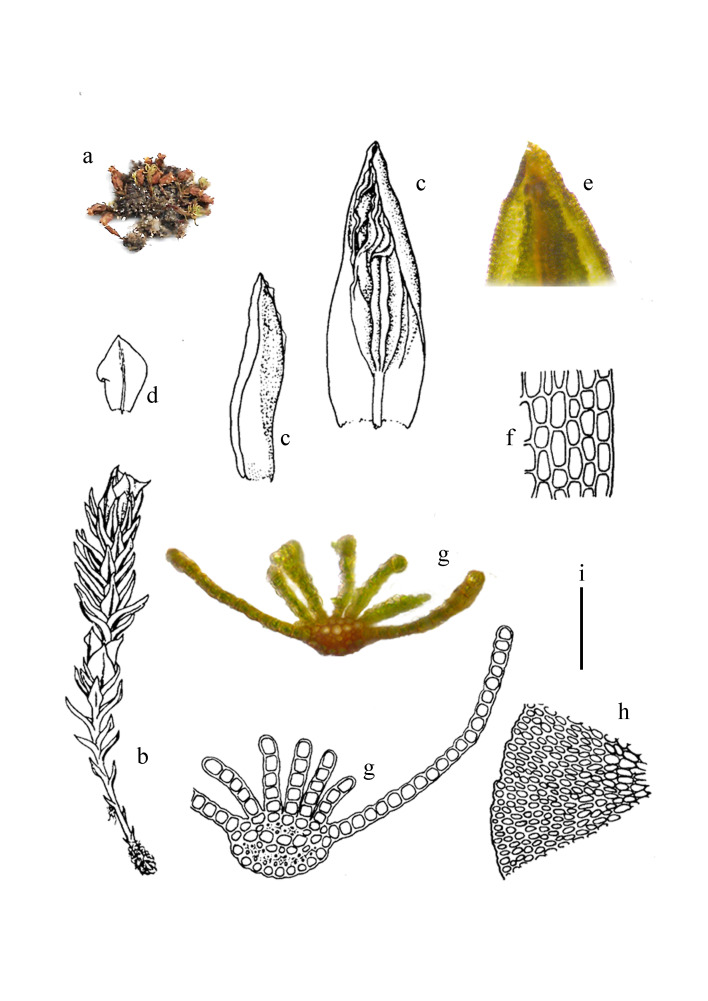
*Oligotrichumobtusatum* Broth. **a** Plant; **b** Moist male plant; **c** Leaves; **d** Perigonial leaves; **e** Apex of leaf (dorsal view); **f** Basal leaf cells; **g** Cross sections of leaf; **h** Portion of the cross section of stem. Scale bars: i = 1 cm (a); i = 2.5 mm (b); i = 1 mm (c, d); i = 200 μm (e, h); a = 100 μm (f, g).

Plants rather small, 0.5-1.2 cm high, brownish green to reddish-brown, often in tufts (Fig. 1a and b). Leaves nearly oblong-oval (Fig. 1c), 1.5-2.0 × 0.5-1.0 mm, usually concave; margins serrate; costa somewhat broad, ending near the apex (Fig. 1e); ventral lamellae slightly wavy or crisped, usually in 3-10 rows, 3-5 cells high; on leaf back with only a few spines (Fig. 1c); median leaf cells rounded quadrate to irregularly oval, 9-15 μm wide; basal cells elongate or irregularly rectangular, 18-25 × 8-16 μm, slightly thick-walled (Fig. 1f-h). Dioecious. Perichaetial leaves lanceolate above, sheathing at base (Fig. 1d). Setae reddish-brown, straight; capsules oblong-ovoid to shortly cylindrical, usually curved; peristome teeth 32; opercula conical, shortly beaked. Calyptrae cucullate, smooth. Spores spherical, ca. 10 μm in diameter, finely papillose (Wu and Jia 2004).

#### Distribution

China (Tibet [present study]; Guizhou, Yunnan and Fujian, India and Nepal (Hyvönen and Lai 1991, Wu and Jia 2004).

### Pogonatum contortum

(Menzies ex Brid.) Lesq.

AAF24359-E33B-5E6E-B4B0-C024CBD283A9

#### Materials

- **Type status:** Other material. **Occurrence:** catalogNumber: PE2155369; recordNumber: 11634; recordedBy: Q. He; occurrenceID: 4CE59F90-8280-55CA-8FDE-83A136F6815C; **Taxon:** order: Polytrichales; family: Polytrichaceae; genus: Pogonatum; **Location:** island: Asia; country: China; countryCode: China/CN; stateProvince: Tibet; county: Cona; municipality: Langpo; locality: Langpo Ditch; verbatimLocality: Shannan; verbatimElevation: 3654 m; verbatimCoordinates: 27º47′55"N, 91º48′51″E; **Identification:** identifiedBy: Y. Jia and Q. He; **Event:** year: 2018; month: 6; day: 18; habitat: On the rocky soil surface; **Record Level:** language: cn
- **Type status:** Other material. **Occurrence:** catalogNumber: PE2155421; recordNumber: 11690; recordedBy: Q. He; occurrenceID: 9FBEF4E6-C16A-5962-B171-F48C9A28973C; **Taxon:** order: Polytrichales; family: Polytrichaceae; genus: Pogonatum; **Location:** island: Asia; country: China; countryCode: China/CN; stateProvince: Tibet; county: Cona; municipality: Mama; locality: Wild Wolf Valley; verbatimLocality: Shannan; verbatimElevation: 3220 m; verbatimCoordinates: 27º52′12"N, 91º59′04″E; **Identification:** identifiedBy: Y. Jia and Q. He; **Event:** year: 2018; month: 6; day: 11; habitat: On the forest floor; **Record Level:** language: cn
- **Type status:** Other material. **Occurrence:** catalogNumber: PE2155455, PE2155456; recordNumber: 11728, 11729; recordedBy: Q. He; occurrenceID: B470F067-EF66-5DD4-B4FC-33C402E71ABD; **Taxon:** order: Polytrichales; family: Polytrichaceae; genus: Pogonatum; **Location:** island: Asia; country: China; countryCode: China/CN; stateProvince: Tibet; county: Cona; municipality: Gongri; verbatimLocality: Shannan; verbatimElevation: 3045-3399 m; verbatimCoordinates: 27º54′36"-55′46"N, 91º48′23″-49′09"E; **Identification:** identifiedBy: Y. Jia and Q. He; **Event:** year: 2018; month: 6; day: 12; habitat: On the roadside soil surface; **Record Level:** language: cn

#### Description

**Figure 2.**
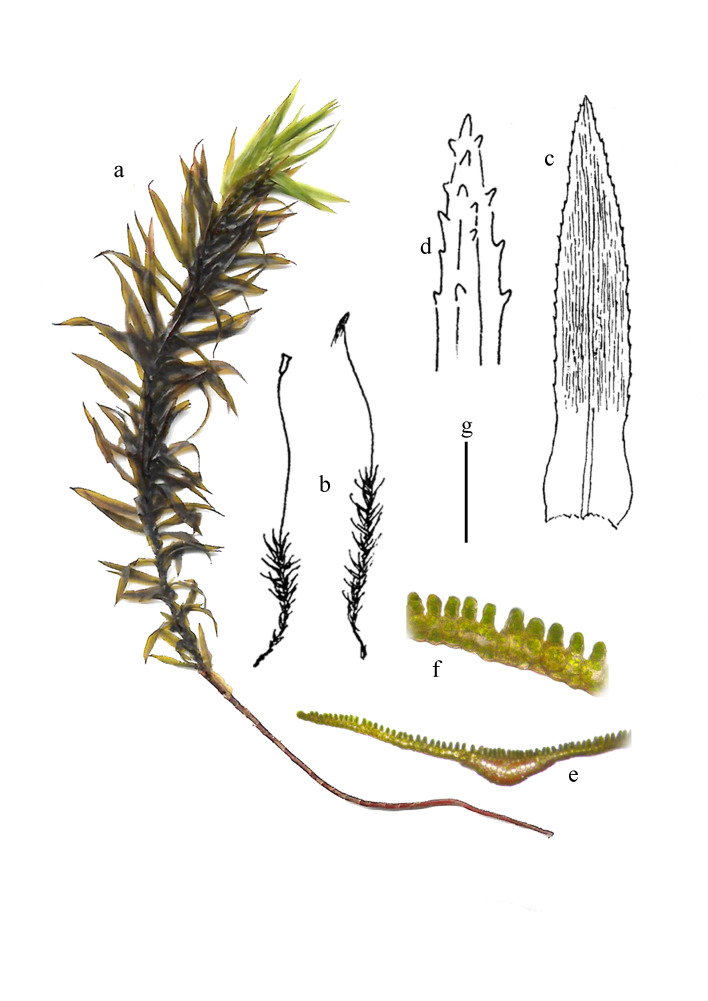
*Pogonatumcontortum* (Menzies ex Brid.) Lesq. **a** Plant; **b** Habit; **c** Leaf (ventral view); **d** Leaf apex; **e** Cross sections of leaf; **f** Part of cross sections of the lamellae of leaf. Scale bars: g = 1 cm (a); g = 2 cm (b); g = 3 mm (c); g = 1 mm (d); g = 200 μm (e); g = 100 μm (f).

Plants large, dark green, brownish-green when old, tufted in large patches (Fig. 2a). Stems 5-10 cm long, usually simple. Basal leaves small, deciduous; upper leaves erect patent when moist, strongly crisped when dry, slightly constricted from rounded oval base to a broad lanceolate limb, sheathing indistinct, ca. 1 mm wide (Fig. 2c); leaf marginal cells 1-2 layers, up to 3 cells wide, rarely remotely grossly toothed, teeth consisting of several cells, light brown; costae broad, with spines on the back; lamellae ca. 40 ranks, 2-3 cells high, disappearing along leaf margins, (Fig. 2e and f); apical cells of lamellae slightly differentiated (Fig. 2d), larger, round, 15-20 μm in diameter, thin-walled; other cells short-quadrate or nearly rectangular, 12-25 × 10-15 μm. Dioecious. Male plants dwarfed; perigonial leaves few. Setae single, dark brown, 20-30 mm long; capsules erect, narrowly ovoid, ca. 2.5 × 1.0 mm (Fig. 2b).

#### Distribution

China (Tibet [present study]; Sichuan, Guangdong, Guangxi, and Hainan), Japan, Russia (Far East), and western North America (Wu and Jia 2004).

### Polytrichum sphaerothecium

(Besch.) Müll. Hal.

8640BD9E-E4C4-503B-9993-EB368912FA22

#### Materials

- **Type status:** Other material. **Occurrence:** catalogNumber: PE2155741; recordNumber: 12035; recordedBy: Q. He; occurrenceID: 6BB4EF1B-4969-55F7-A776-DD969F0109CE; **Taxon:** order: Polytrichales; family: Polytrichaceae; genus: Polytrichum; **Location:** island: Asia; country: China; countryCode: China/CN; stateProvince: Tibet; county: Dingri; municipality: Qudang; locality: Cuoqu; verbatimLocality: Shigatse; verbatimElevation: 3796 m; verbatimCoordinates: 28º05′23"N, 87º21′53"E; **Identification:** identifiedBy: Y. Jia and Q. He; **Event:** year: 2018; month: 6; day: 21; habitat: On the forest floor; **Record Level:** language: cn

#### Description

**Figure 3.**
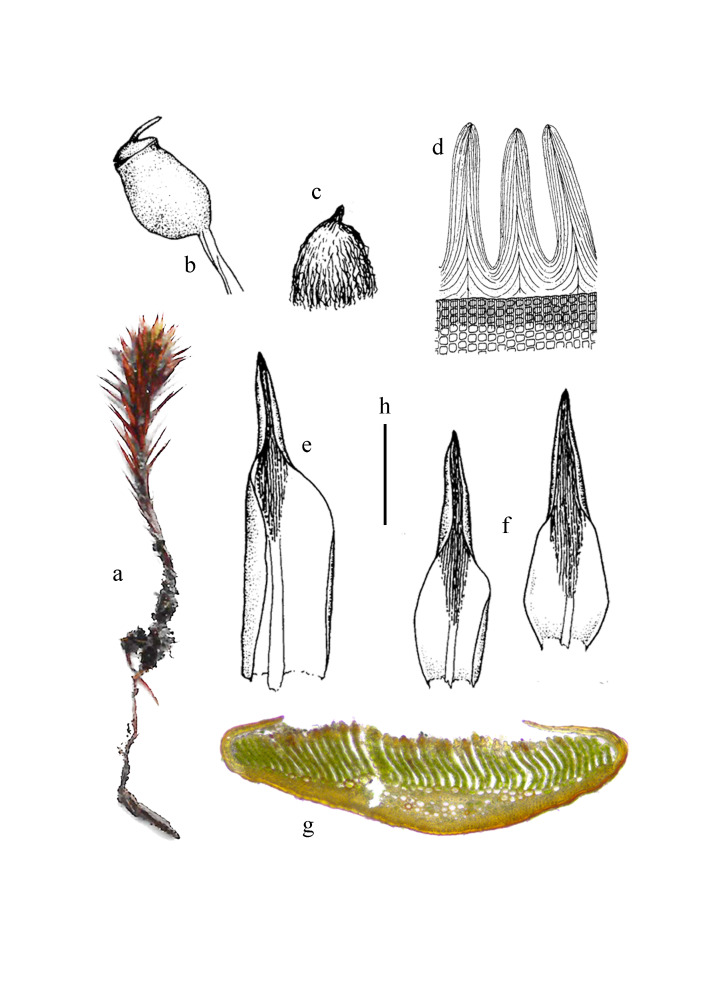
*Polytrichumsphaerothecium* (Besch.) Müll. Hal. **a** Plant; **b** Capsule; **c** Calyptra; **d** Peristome teeth; **e** Outer perichaetial leaf; **f** Leaves; **g** Cross section of leaf. Scale bars: h = 1 cm (a); h = 3 mm (b, c); h = 200 μm (d, g); h = 2 mm (e, f).

Plants small, brownish-green, slightly glossy, in dense aggregated tufts (Fig. 3a). Stems erect, 1-3 cm high. Leaves slightly incurved, usually appressed on stems when dry, erect-patent when moist, 3-6 × 1.2-1.5 mm, ovate-lanceolate to oblong-lanceolate, acuminate or cucullate at apex, sheathing indistinct (Fig. 3f, g); costa excurrent into a short filiform apex, smooth or a few teeth on back near apex; leaf margins involute, covering ventral lamellae; upper leaf cells quadrate or oblate, 12-15 μm wide, sometimes the length 1-2 times longer than width; median cells rectangular, 18-40 × 8-10 μm; basal cells quadrate to narrowly rectangular, bordered by narrow, hyaline cells; ventral lamellae in ca. 30 rows, 6-11 cells high; marginal cells of lamellae pyriform, thick-walled. Dioecious. Male plants similar to the female ones, occasionally with new innovations. Perigonial leaves ca. 3 mm long, with oblong sheathing base. Perichaetial leaves up to 9 mm long, from an ovate sheathing base, suddenly narrowing to a lanceolate lamina (Fig. 3e). Setae 5-9 mm long, pendulous; capsules nearly spherical, horizontal to cernuous, with distinct discoid apophyses (Fig. 3b); exothecial cells hexagonal to triangular, without mammillae; opercula with short beaks; peristome teeth often more than 32, triangular, acuminate, ca. 30 μm long (Fig. 3d), greyish-yellow, membrane nearly as long as the peristome teeth. Calyptrae triangular, 3-4 mm long, densely covered with long hairs (Fig. 3c).

#### Distribution

China (Tibet [present study], Jilin), Korea, Japan and the Aleutian Islands (Wu and Jia 2004).

## Checklists

### A checklist of the Polytrichaceae in Tibet, China

#### Atrichum crispulum

Schimp. ex Besch.

89337418-D932-531F-8991-B19A10114659

##### Distribution

China, North Korea, Japan and Thailand (Wu and Jia 2004).

#### Atrichum rhystophyllum

(Müll. Hal.) Paris

3537D5A9-60C3-5257-9E23-4DDFC90839CC

##### Distribution

China, North Korea and Japan (Wu and Jia 2004).

#### Atrichum undulatum var. gracilisetum

Besch.

AB6E3BDF-AC40-5CB0-8F38-2EE3D2BF393D

##### Distribution

China, Pakistan (Higuchi and Nishimura 2003), Myanmar (Tan and lwatsuki 1993), North Korea, Japan and the Himalayan Region (Wang et al. 2018).

#### Lyellia crispa

R. Br.

BC3FBD6E-EFEC-5CE8-83A9-584357EADC8E

##### Distribution

China, the Eastern Himalayas and North America (Wu and Jia 2004).

#### Lyellia platycarpa

Cardot & Thér.

3A25E1BE-E102-5153-A2A0-5CBBDFE335B7

##### Distribution

Endemic species of China (Wu and Jia 2004).

#### Oligotrichum aligerum

Mitt.

6C3311A1-0D2C-5F18-9AB7-3B463A82981C

##### Distribution

China, Bhutan, Japan, Korea, North America, Central America (Wang et al. 2018).

#### Oligotrichum crossidioides

P.C. Chen & T.L. Wan ex W.X. Xu & R.L. Xiong

BF1CA4A1-91EE-5905-A750-4344C642BE33

##### Distribution

Endemic to China, occurring in the south-western part of the country, as well as in the Himalayan Region (Wu and Jia 2004).

#### Oligotrichum falcatum

Steere

A0619113-FEAD-507C-9530-C907ACDF9E95

##### Distribution

China (Tibet), the Himalayan Region, Russia (Siberia), the United States (Alaska), Canada and Greenland (Wu and Jia 2004).

#### Oligotrichum hercynicum

(Hedw.) DC.

47E3D986-E0CC-5AE7-937F-8B75060B4287

##### Distribution

China (Tibet) (Wu and Jia 2004), Japan, Europe and North America (Merrill 2007).

#### Oligotrichum obtusatum

G.L. Sm.

F3822466-1D8A-5D5B-81FB-9A5C23B20E2D

##### Distribution

Jilong Township, Jilong County and Gongri Township, Cona County.

##### Notes

China (Tibet [present study]; Guizhou, Yunnan and Fujian (Wu and Jia 2004) and Nepal (Hyvönen and Lai 1991).

#### Oligotrichum semilamellatum

(Hook. f.) Mitt.

4C8A5707-D952-5CC9-AE08-FEF98534F1EF

##### Distribution

Southwest China and the Himalayan Region (Wu and Jia 2004).

#### Pogonatum aloides

(Hedw.) P. Beauv.

9F534358-06ED-5A2E-B466-B366FBAD2188

##### Distribution

China, Nepal, Bhutan, India, Sri Lanka, Myanmar (Tan and lwatsuki 1993), Thailand, Vietnam, Philippines, Indonesia, Japan, Caucasus, North America, North and Central Africa (Wu and Jia 2004).

#### Pogonatum cirratum fuscatum

(Mitt.) Hyvönen

F41F9F03-ADC3-555B-A46B-81023591A938

##### Distribution

China (Wu and Jia 2004), Bangladesh (O'Shea 2003), Nepal, Bhutan, India, Myanmar, Laos (Hyvönen and Lai 1991), Vietnam, Malaysia, Philippines, Chile (He 1998).

#### Pogonatum contortum

(Menzies ex Brid.) Lesq.

C33E9535-E1F5-5F3A-83DC-700F8DDCC47B

##### Distribution

Mama Township, Langpo Township, Gongri Township, Cona County.

##### Notes

China (Tibet [present study]; Sichuan, Guangdong, Guangxi and Hainan), Japan, Russia (Far East) and western North America (Wu and Jia 2004).

#### Pogonatum inflexum

(Lindb.) Sande Lac.

EE99FFCB-76E4-5EDD-891E-2DB3D2033EA2

##### Distribution

China, North Korea and Japan (Wu and Jia 2004).

#### Pogonatum microstomum

(R. Br. ex Schwägr.) Brid.

CA4B3178-3740-58B3-8A0B-684DC58ED7BC

##### Distribution

Himalayan Region of China and South Asia (Wu and Jia 2004).

#### Pogonatum nudiusculum

Mitt.

EB42552D-1C97-57F7-9371-A4C123863E22

##### Distribution

China, Nepal, Bhutan, India, Sri Lanka and the Philippines (Wu and Jia 2004).

#### Pogonatum pergranulatum

P.C. Chen

84096282-C15C-542D-8E07-6232D4E5CBF7

##### Distribution

Endemic species of China (Wu and Jia 2004).

#### Pogonatum perichaetiale

(Mont.) A. Jaeger

7C07D9C1-8373-5FAF-9DDD-94FD5F86374D

##### Distribution

China, Nepal, Sikkim, Bhutan and southern India (Wu and Jia 2004).

#### Pogonatum sinense

(Broth.) Hyvönen & P.C. Wu

2D082E0E-DCEB-50E3-9D2A-39634813D7B0

##### Distribution

China and Bhutan (Wang et al. 2018).

#### Pogonatum subfuscatum

Broth.

505F7BBF-8553-5456-B7F0-442FE218D3DB

##### Distribution

Widely distributed in southern Asia (Wu and Jia 2004).

#### Pogonatum urnigerum

(Hedw.) P. Beauv.

60D38430-0CF0-56F5-87CD-B927D250CC9E

##### Distribution

Widespread in the Northern Hemisphere, but not in high-altitude mountains (Wu and Jia 2004).

#### Polytrichastrum alpinum

(Hedw.) G.L. Sm.

62D0B16C-E072-5E34-B462-F2C6CFFE706E

##### Distribution

Widely distributed throughout the world (Wang et al. 2018).

#### Polytrichastrum emodi

G.L. Sm.

B80F7471-221C-5064-A30A-B03F46F820C1

##### Distribution

China (south-western region) and the Himalayan Region (Wu and Jia 2004).

#### Polytrichastrum formosum var. formosum

(Hedw.) G.L. Sm.

79A3B883-15F3-507D-929D-1C7854F8458B

##### Distribution

China, the Himalayan Region, Japan, Russia (Far East), Europe, North Africa, the Aleutian Islands and North America (Wu and Jia 2004).

#### Polytrichastrum longisetum

(Sw. ex Brid.) G.L. Sm.

03019331-5EC3-56B0-944E-E35F082C8954

##### Distribution

China, Japan, Russia (Siberia), Europe, Greenland, North America and New Zealand (Wu and Jia 2004).

#### Polytrichastrum papillatum

G.L. Sm.

D4309323-30E9-52DC-BFFC-B68B306F2391

##### Distribution

China (Tibet) and the Himalayan Region (Wu and Jia 2004).

#### Polytrichastrum xanthopilum

(Wilson ex Mitt.) G.L. Sm.

FEEC9B8B-3067-5C59-A802-A153E6D88AAE

##### Distribution

China, the Himalayan Region and North America (Wu and Jia 2004).

#### Polytrichum juniperinum

Hedw.

14FE89E2-689E-5E2C-A431-2AC49862F13A

##### Distribution

It is widespread in northeast and southwest China and Xinjiang, Japan, Russia (Far East), Europe, North and South America and Oceania (Li 1985).

#### Polytrichum piliferum

Hedw.

FB5AB0ED-0CAB-5CCB-8650-FE6A2B951C61

##### Distribution

China, Korea, Japan, Russia (Siberia, Sakhalin and Kuril Islands), Chile, Europe, North America, Africa (Wu and Jia 2004).

#### Polytrichum sphaerothecium

(Besch.) Müll. Hal.

CB3359B6-47D7-5E66-A91F-13596B0D0EF4

##### Distribution

Qudang Township, Dingri County.

##### Notes

China (Tibet [present study], Jilin), Korea, Japan and the Aleutian Islands (Wu and Jia 2004).

## Discussion

The present study reveals substantial novel insights pertaining to the family Polytrichaceae in Tibet, China, thereby contributing to the progressive refinement of our comprehension of this diverse group of mosses. The discovery and subsequent documentation of three previously-undocumented species — *Oligotrichumobtusatum*, *Polytrichumsphaerothecium* and *Pogonatumcontortum* — not only accentuate the biological richness, but also underscore the existing deficiencies in our taxonomic and ecological understanding of the region.

*Oligotrichumobtusatum*, designated as Vulnerable (VU) by the Red List of Higher Plants in China (Qin et al. 2017), necessitates significant conservation attention. The species' occurrence in Tibet expands its previously documented geographic range, which was limited to regions including the Russia (Far East) and western North America (Wu and Jia 2004). This discovery warrants further investigation into the dispersal mechanisms and habitat requirements of *O.obtusatum*, especially considering the distinctive climatic and topographical conditions characteristic of Tibet.

Moreover, the documentation of *Polytrichumsphaerothecium*, a species previously recorded exclusively in Jilin (China) and classified as Data Deficient (DD) (Qin et al. 2017), enriches our biological inventories and prompts further ecological and phylogenetic research to elucidate its restricted distribution and ecological preferences. *Pogonatumcontortum* is known to be distributed across southwest China, the Russia (Far East), Japan and western North America (Wu and Jia 2004). Notably, it is not presently listed on the List (Qin et al. 2017). The current discovery of the species in Tibet further extends its documented geographical range.

This study significantly enhances the documentation of regional biodiversity by updating the enumeration of Polytrichaceae in Tibet to include six genera and 28 species, one subspecies and two varieties, which not only offers a more comprehensive understanding of the Tibetan bryoflora, but also establishes a critical reference point for future research aimed at taxonomic clarification and conservation initiatives. Continued exploration and detailed study are essential for the conservation of biodiversity and the sustainability of natural resources in the high-altitude ecosystems.

## Supplementary Material

XML Treatment for Oligotrichum obtusatum

XML Treatment for Pogonatum contortum

XML Treatment for Polytrichum sphaerothecium

XML Treatment for Atrichum crispulum

XML Treatment for Atrichum rhystophyllum

XML Treatment for Atrichum undulatum var. gracilisetum

XML Treatment for Lyellia crispa

XML Treatment for Lyellia platycarpa

XML Treatment for Oligotrichum aligerum

XML Treatment for Oligotrichum crossidioides

XML Treatment for Oligotrichum falcatum

XML Treatment for Oligotrichum hercynicum

XML Treatment for Oligotrichum obtusatum

XML Treatment for Oligotrichum semilamellatum

XML Treatment for Pogonatum aloides

XML Treatment for Pogonatum cirratum fuscatum

XML Treatment for Pogonatum contortum

XML Treatment for Pogonatum inflexum

XML Treatment for Pogonatum microstomum

XML Treatment for Pogonatum nudiusculum

XML Treatment for Pogonatum pergranulatum

XML Treatment for Pogonatum perichaetiale

XML Treatment for Pogonatum sinense

XML Treatment for Pogonatum subfuscatum

XML Treatment for Pogonatum urnigerum

XML Treatment for Polytrichastrum alpinum

XML Treatment for Polytrichastrum emodi

XML Treatment for Polytrichastrum formosum var. formosum

XML Treatment for Polytrichastrum longisetum

XML Treatment for Polytrichastrum papillatum

XML Treatment for Polytrichastrum xanthopilum

XML Treatment for Polytrichum juniperinum

XML Treatment for Polytrichum piliferum

XML Treatment for Polytrichum sphaerothecium
